# Characterization of Ozonation Transformation Products of Flutamide and Azathioprine by UHPLC-Ion Mobility-High-Resolution Mass Spectrometry

**DOI:** 10.1021/acsestwater.5c01314

**Published:** 2026-03-24

**Authors:** Josep García-Martínez, Cristina Corpa, Guillermo Nieto, María Concepción Monte, Přemysl Soldán, Ángeles Blanco, Peter Spégel

**Affiliations:** † Department of Chemistry, Centre for Analysis and Synthesis, Lund University, P.O. Box 124, Lund 22100, Sweden; ‡ Department of Chemical Engineering and Materials, 16734 Universidad Complutense of Madrid, Avda. Complutense s/n, Madrid 28040, Spain; § T. G. Masaryk Water Research Institute, Podbabská 2582/30, Prague 16000, Czech Republic

**Keywords:** ozonation, anticancer pharmaceuticals, transformation products, non-target analysis, toxicity

## Abstract

Ozonation is one of the most promising advanced oxidation processes to be implemented as quaternary treatment for the removal of micropollutants in wastewater treatment plants. However, there are some knowledge gaps that limit its implementation. First, the removal efficiency is not known for all pollutants, and second, knowledge about degradation byproducts is limited, as well as the toxicity of the treated waters. In this work, the removal efficiency of azathioprine and flutamide is studied, as well as the transformation products formed during ozonation. A lab-scale reactor was used for ozonation of the drugs in pure water, with ambient air as the source for ozone generation and liquid chromatography–ion mobility-high-resolution mass spectrometry for time-resolved process monitoring. Notably, azathioprine and flutamide were not completely removed by ozonation, with 49–54% remaining after the treatment. Furthermore, two transformation products of azathioprine and three of flutamide were identified, including previously unreported nitroaromatic transformation products of flutamide. While both parent compounds showed relatively low predicted toxicity, transformation products of flutamide exhibited higher predicted chronic toxicity. These results highlight the importance of considering transformation products during ozonation and suggest possible nitration reactions when ozone is generated from ambient air. Further experiments are needed to confirm this.

## Graphical abstract

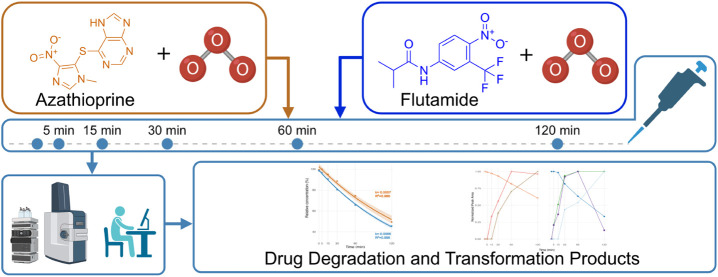

## Introduction

Advanced oxidation processes (AOPs) are a set of water treatments designed to remove organic pollutants by direct oxidation or by generating reactive species such as hydroxyl radicals, which can rapidly degrade organic compounds.^1^ The goal of AOPs is to reduce the emission of pollutants by transforming them into simpler, less harmful products.^2^ AOPs are effective treatments for the removal of pharmaceuticals in wastewater.^3,4^ Among the AOPs, ozonation is one of the most promising techniques. On the one hand, it is a consolidated technology implemented at a large scale for disinfection, deodorization, increasing water biodegradability or removal of recalcitrant organic material.^5,6^ On the other hand, it exploits the strong oxidizing potential of ozone (1.03 V; direct ozonation) and the even greater oxidizing potential of hydroxyl radicals (2.31 V; indirect ozonation).^7^ Although both mechanisms may coexist depending on several factors, such as ozone dose, chemical properties of the contaminant, amount and type of organic matter, and the presence of radical scavengers, under near-neutral pH as in the case of urban wastewater, direct molecular ozone pathway predominates.^8^ While quaternary treatment based on ozonation holds considerable potential for fulfilling the EU Directive 2024/3019 on urban wastewater treatment, its implementation remains limited by critical knowledge gaps regarding removal efficiency, degradation pathways and toxicity of the treated water.

Common analytical methods are often targeted toward a set of known compounds, overlooking the broad spectrum of transformation products (TPs) formed during AOPs. Nontargeted analysis using high-resolution mass spectrometry (HRMS) has become increasingly utilized to characterize these TPs,^9,10^ providing insights into their chemical structures and formation pathways, information which can be used to assess their environmental impact. This holistic characterization is crucial, as some TPs may exhibit increased toxicity or persistence compared to their parent compounds.^11^

Azathioprine (AZA) is an immunosuppressive drug commonly prescribed for autoimmune disorders such as inflammatory bowel disease.^12^ After administration, AZA is converted into 6-mercaptopurine, which interferes with DNA synthesis.^13^ It is estimated that in Spain, more than 20.000 patients per year are treated with AZA.^14^ Flutamide (FLUT) is a drug primarily used in the treatment of prostate cancer. In Spain, the annual consumption of FLUT was estimated at approximately 1987 kg/year,^15,16^ primarily through pharmacy distribution, with around 10% of the parent compound excreted unchanged.^17^ Although both AZA and FLUT are metabolized in humans, they are still detected in wastewater^18^ and represent relevant targets for AOPs.

AZA and FLUT have been detected in Spanish municipal and hospital wastewater.^18^ AZA concentrations in hospital wastewaters range from 9 to 90 ng/L,^18,19^ while its presence in municipal WWTPs range 19–20 ng/L in influents to <1.8 ng/L in effluents.^18^ FLUT concentrations in hospital wastewater range from 2.1 to 6.2 ng/L^20^ and has been detected in the aquatic environment at ng/L.^21^ These findings indicate the incomplete removal of these pharmaceuticals during conventional wastewater treatment. According to EU Commission Decision 2000/532/EC251, which established the European List of Waste, they are classified as hazardous waste and pose a significant risk to the aquatic environment, even at very low concentrations.^19,22,23^

For AZA, research has been conducted to investigate the UV and H_2_O_2_-based degradation of 6-mercaptopurine.^17^ In the case of FLUT, some TPs produced in the solar photo-Fenton process have been characterized.^24^ However, to the best of our knowledge, ozonation-derived TPs of AZA and FLUT are yet to be investigated.

This study seeks to assess the effectiveness of ozonation on FLUT and AZA removal, while at the same time aiming to assign chemical structures to the generated TPs and to assess the final potential toxicity of the treated waters.

## Materials and Methods

### Ozonation

Ozonation was conducted using a commercial ozone generator, model ZHI10000 (ASP Asepsia, Madrid, Spain), producing ozone from ambient air with an output of 100 mg O_3_/h and a flow rate of 8 L/min. The ozone was delivered via a porous plate at the base of a 1L glass column containing a solution of FLUT or AZA at 1 mg/L in ultrapure water (Milli-Q water). Stock solutions of AZA and FLUT were prepared in methanol and subsequently diluted with ultrapure water to obtain the working solutions. FLUT and AZA reference standards were purchased from Sigma-Aldrich (St. Louis, MO) with a purity of ≥98%. Kinetics of FLUT and AZA degradation were estimated, and TPs identified, from 1 mL samples collected with a 1 mL syringe at 0, 5, 15, 30, 60, and 120 min. Collected samples were filtered through a 0.22 μm PTFE syringe filter (Agilent Technologies, Santa Clara, CA) and stored at −20 °C until analysis. Duplicate samples, packed in ice, were sent from Madrid to Lund for identification of potential TPs. Blank samples were produced by ozonation of ultrapure water before each oxidation experiment.

### Targeted Analysis

The concentrations of AZA and FLUT were determined on an Agilent 1290 Infinity UHPLC system, equipped with a Poroshell 120 EC-C18 column (50 × 3 mm × 2.7 μm) fitted with a guard column (UHPLC Guard), and coupled to an Agilent 6495C triple quadrupole, all from Agilent Technologies (Santa Clara, CA). Acetonitrile (ACN) (B) and ultrapure water (A) (both with 0.1% formic acid) were obtained from Supelco (LiChrosolv hypergrade, Darmstadt, Germany), suitable for LC-MS applications. The mobile phase gradient started at 5% (B), increased to 100% (B) over 18.5 min, remained at 100% (B) for 1.5 min, and returned to 5% (B) at 24 min. The flow rate was set at 0.6 mL/min, with an injection volume of 20 μL and column temperature of 40 °C. AZA and FLUT were quantified in dynamic multiple-reaction monitoring mode. The most intense transitions were used for quantification (FLUT, 275.1 → 205, 21 V; AZA, 278.0 → 142.1, 9 V) and one additional transition for each analyte were included as qualifiers (FLUT, 275.1 → 202, 29 V; AZA, 278.0 → 231.9, 13 V). The method detection limits were 2.8 and 0.6 ng/L for AZA and FLUT, respectively. Method validation was performed previously and is described elsewhere.^100^

### Nontargeted Analysis

The samples were separated using an Agilent 1290 Infinity UHPLC system (Agilent Technologies) equipped with an Acquity Premier HSS T3 C18 column (100 × 2.1 mm × 1.8 μm, Waters Corporation, Milford, CT). Mobile phase A consisted of ultrapure water, and B was ACN (LC-MS grade, Fisher Scientific, Sweden), both with 0.1% formic acid (LC-MS grade, Fisher Scientific). The flow rate was 0.4 mL/min, and the gradient profile was: 0–1 min, 5% B; 1–11 min, 5–99% B; 11–15 min, 99% B; 15–15.5, 99–5% B; 15.5–18, 5% B. Injection volume was 5 μL and the column temperature 40 °C. The UHPLC was coupled to a timsTOF Pro 2 (Bruker Daltonics, Bremen, Germany) mass spectrometer operating in Parallel Accumulation Serial Fragmentation (PASEF) mode. A 6-port valve was used to supply a mixture of Agilent ESI-Low concentration Mix (Agilent Technologies) and sodium formate for 0.5 min during each analysis for calibration of mass to charge (*m*/*z*) ratio and collision cross section (CCS), respectively. Samples were injected in triplicates followed by an instrumental blank (ultrapure water) after each triplicate. Data were acquired in both positive and negative electrospray ionization mode. Before the analysis, the instrument was thoroughly flushed with ultrapure water, methanol and ACN (both LC-MS grade, Fisher Scientific). Three instrumental blanks were acquired at the start of the run.

To ensure high data quality and minimize the risk of false positives, the study incorporated multiple types of blanks into its design, efficiently eliminating signals unrelated to the samples.^26^ Instrumental blanks were used for the subtraction of background signals originating from the instrumentation itself, while postsample blanks allowed for monitoring of background fluctuation and carry-over effects. Ozonized pure water accounted for potential artifacts introduced by the ozonation equipment, sample handling, and transportation.

### Data Analysis

Raw data were processed using MetaboScape 2022b (Bruker Daltonics). Features, defined as distinct signals characterized by a specific *m*/*z*, retention time (RT), and CCS, were filtered using two criteria: (i) peak area greater than 1.3 times the peak area in the blanks and (ii) variation greater than 20% relative standard deviations between unique samples. The rationale for the second criterion was that the concentration of a TP is expected to change during the ozonation process. After data filtration, features were prioritized for structural evaluation based on their dynamic changes during the 120 min ozonation, with those showing a continuous increase given top priority.

Finally, prioritized features were annotated using the NORMAN Suspect List Exchange (DOI: http://10.5281/zenodo.2664077) and MS libraries such as MassBank of North America (MoNA) and MassBank Europe. SmartFormula, integrated in MetaboScape, was used to generate a list of candidate molecular formulas, which were then submitted to Compound Crawler, also integrated in MetaboScape, to retrieve candidate structures from PubChem and ChemSpider. These structures were further evaluated in MetaboScape using MetFrag^27^ and CCS Predict Pro^28^ for in silico MS/MS fragmentation and CCS prediction, respectively. Additionally, data were exported to SIRIUS (version 6.2) for molecular formula annotation,^29^ chemical class prediction via CANOPUS,^30^ and de novo structure generation based on MS/MS fragmentation patterns.^31^

### Toxicity Assessment

The ecotoxicity of the samples prior to and post treatment was assessed using a combination of experimental and predictive methods. The acute toxicity of the samples was evaluated using a test with luminous bacteria (*Aliivibrio fischeri*) carried out according to ISO 11348-3:2009. This assay is a rapid, simple, and widely accepted screening method.^32^ Measurements were carried out using a Biolight Toxy equipment from Aqua Science (Columbus, OH). Samples were filtered (0.22 μm PTFE syringe filter, Agilent Technologies), pH-adjusted to 7 using 0.1 M HCl and 1 M NaOH, and exposed to the bacteria for 15 min at 15 °C. Luminescent bacteria tests were performed on solutions containing 1 mg/L AZA or FLUT before ozonation and also on solutions after 120 min of ozonation. In accordance with the standard test procedure, the range of concentrations tested included a maximum possible concentration of 45% and ended with a concentration of 5% of the original sample.

Additionally, the Ecological Structure Activity Relationships (ECOSAR v2.2, U.S. Environmental Protection Agency) predictive model, was used to estimate possible acute and chronic aquatic toxicity, based on chemical structures represented by compound Simplified Molecular Input Line Entry System (SMILES). ECOSAR provided estimated LC_50_, EC_50_, and chronic values (ChV) for fish, Daphnia, and algae.

## Results and Discussion

### Oxidation of FLUT and AZA

After application of a cumulative ozone dose of 200 mg O_3_/L, both FLUT and AZA remained detectable at approximately 50% of their initial concentrations (Figure 1). Under typical wastewater treatment conditions, where lower ozone doses are applied, the removal of these target compounds would be limited (<10% for 25 mg O_3_/L or <20% for 50 mg O_3_/L).

**Figure 1:**
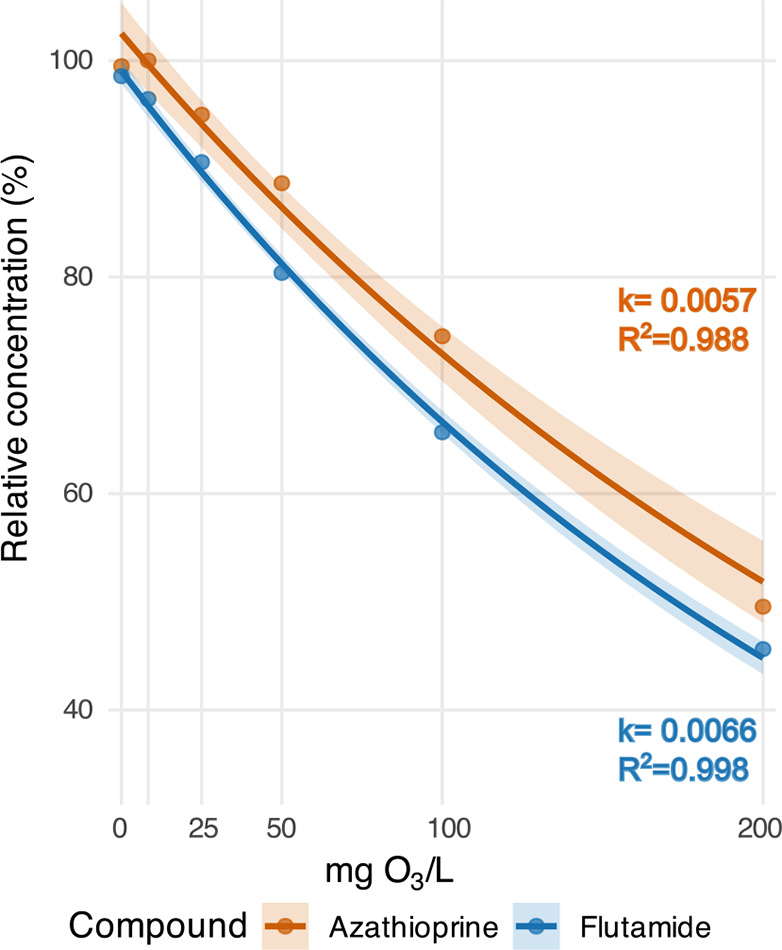
Oxidation of azathioprine and flutamide during ozonation in ultrapure water (initial concentration: 1 mg/L). Solid lines show fits to a pseudo-first-order kinetic model, and the shaded areas represent the 95% confidence intervals of the fits. The corresponding apparent rate constants (k) are provided in the figure.

Removal rates for both AZA and FLUT followed a pseudo-first-order equation, with kinetic constants of 5.7·10^–3^ min^–1^ and 6.6·10^–3^ min^–1^, respectively. Coefficients of determination (R^2^) for both models were above 0.98. Hence, degradation through ozonation was compound-dependent. The ozonation experiments were performed at approximately neutral pH, where ozone acts as the primary oxidant rather than hydroxyl radicals.^33^ However, to prove this, quencher experiments would be needed, which were not conducted in the present investigation. While the applied conditions allow investigation of ozone-driven transformation pathways, ozonation in real wastewater, where the concentration and composition of organic matter differ, may result in different removal rates and TPs. High ozone dosages were applied to ensure compound removal and to assess whether the TPs were stable or quickly degraded under ozonation.

### Identification of TPs

To monitor the formation and evolution of TPs during ozonation, aliquots were collected at various time points throughout the oxidation process. By using this strategy, both labile and persistent TPs could be monitored. Furthermore, this approach supports a process-driven prioritization of features, consistent with a nontargeted analysis workflow where TPs are initially treated as unknowns.^34^

Only features consistently present in all three replicate injections were retained to eliminate artifacts from electronic noise, carry-over, or other random sources. Furthermore, features with low peak area variations across all time points, including the unoxidized sample, were excluded, as they were unlikely to represent TPs. The number and percentage of excluded features by blanks are presented in Figure 2A and B together with examples of features removed by blanks (Figure 2C,D and E). This exemplifies the importance of including several types of blanks for this kind of investigation.

**Figure 2:**
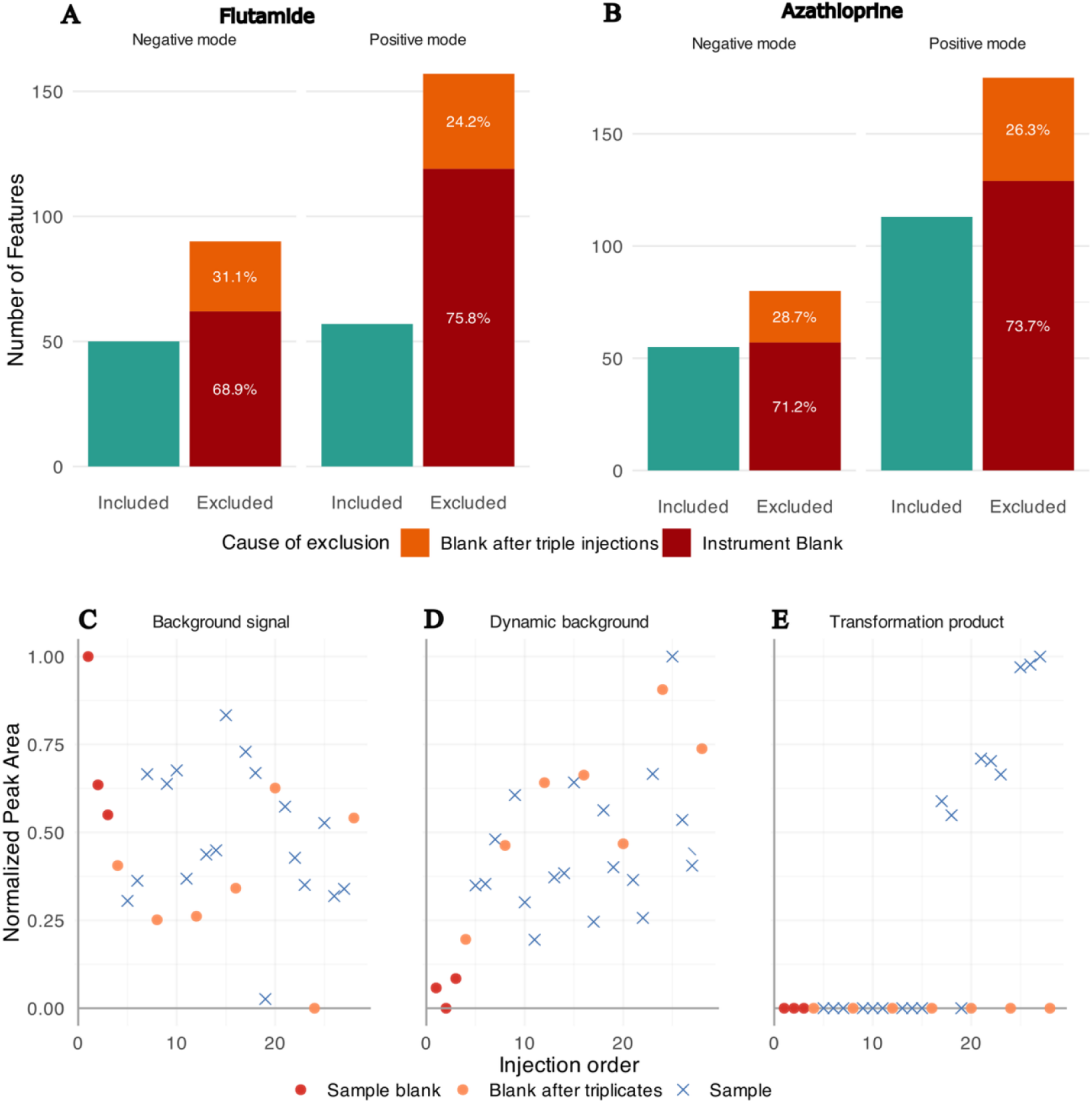
Number of features excluded and included for the experiments for (A) flutamide and (B) azathioprine. Examples illustrating the feature prioritization workflow: (C) Feature detected with similar intensity in blanks and samples, excluded as background. (D) Feature present in initial blanks and increasing over time but also detected in blanks between samples, excluded as nonconsistent background. (E) Feature absent from blanks and showing time-dependent formation during ozonation, selected as a transformation product.

Feature selection was performed by first excluding signals present in blanks and background controls. Features that were detected in blanks with similar intensity as in samples, or that varied over time but were also present in blanks collected after triplicate injections of samples, were not considered TPs. Only features that were absent in all blanks, showed consistent, time-dependent formation during ozonation and provided MS/MS data were kept.

Following data prioritization, two TP candidates remained for AZA and three for FLUT (Figure 3). Whereas both FLUT and AZA were successfully annotated using the MS/MS libraries and the NORMAN target list, these libraries failed to identify any of the detected TPs. This indicates that there is extensive work ahead to characterize relevant TPs and include them in databases even when labeled as tentative structure.

**Figure 3:**
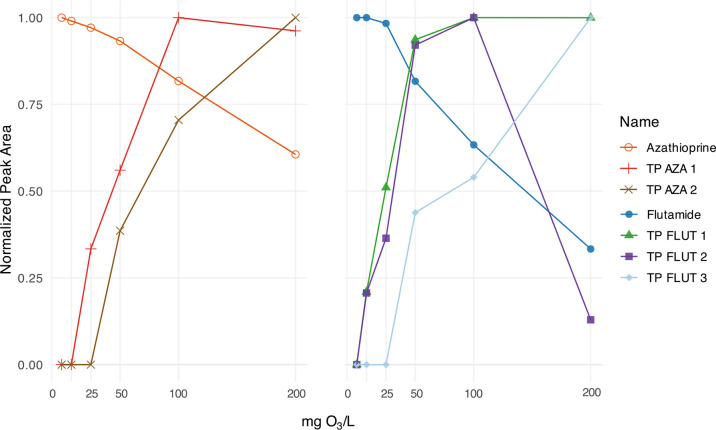
Alterations in azathioprine (AZA), flutamide (FLUT) and their transformation products (TPs) over the 120 min ozonation process. Data are presented as peak areas normalized to the highest peak area for the indicated compound.

Candidates for each TP were ranked based on their predicted MS/MS spectral coverage, CCS values and chemical class prediction. Additionally, chemical expertise was applied to prioritize plausible structures. For example, in FLUT, the trifluoromethyl (−CF_3_) group was not expected to migrate from the aromatic ring to the aliphatic chain, helping to rule out several unlikely candidates. Molecular formulas, CCS values, and RTs for the TPs are found in Table 1, and their suggested structures are shown in Figure 4.

**Table 1:** Information on the Studied Features

Name	Mass Measured	Molecular Formula	Error (ppm)^a^	RT (min)	CCS	CCS (predicted)^a^
Azathioprine	277.03877	C_9_H_7_N_7_O_2_S	2.10	4.12	154.4	153.5
TP AZA 1	293.03343	C_9_H_7_N_7_O_3_S	1.39	3.64	154.9	154.4
TP AZA 2	200.00027	C_5_H_4_N_4_O_3_S	–0.58	0.86	131.6	130.6
Flutamide	276.07252	C_11_H_11_F_3_N_2_O_3_	1.74	8.61	154.0	151.5
TP FLUT 1	251.01562	C_7_H_4_F_3_N_3_O_4_	–1.42	7.83	137.9	138.4
TP FLUT 2	251.01544	C_7_H_4_F_3_N_3_O_4_	–0.06	7.97	135.1	135.4
TP FLUT 3	321.05751	C_11_H_10_F_3_N_3_O_5_	–0.06	8.60	159.3	159.0

a In relation to the proposed structure, see Figure 4.

**Figure 4:**
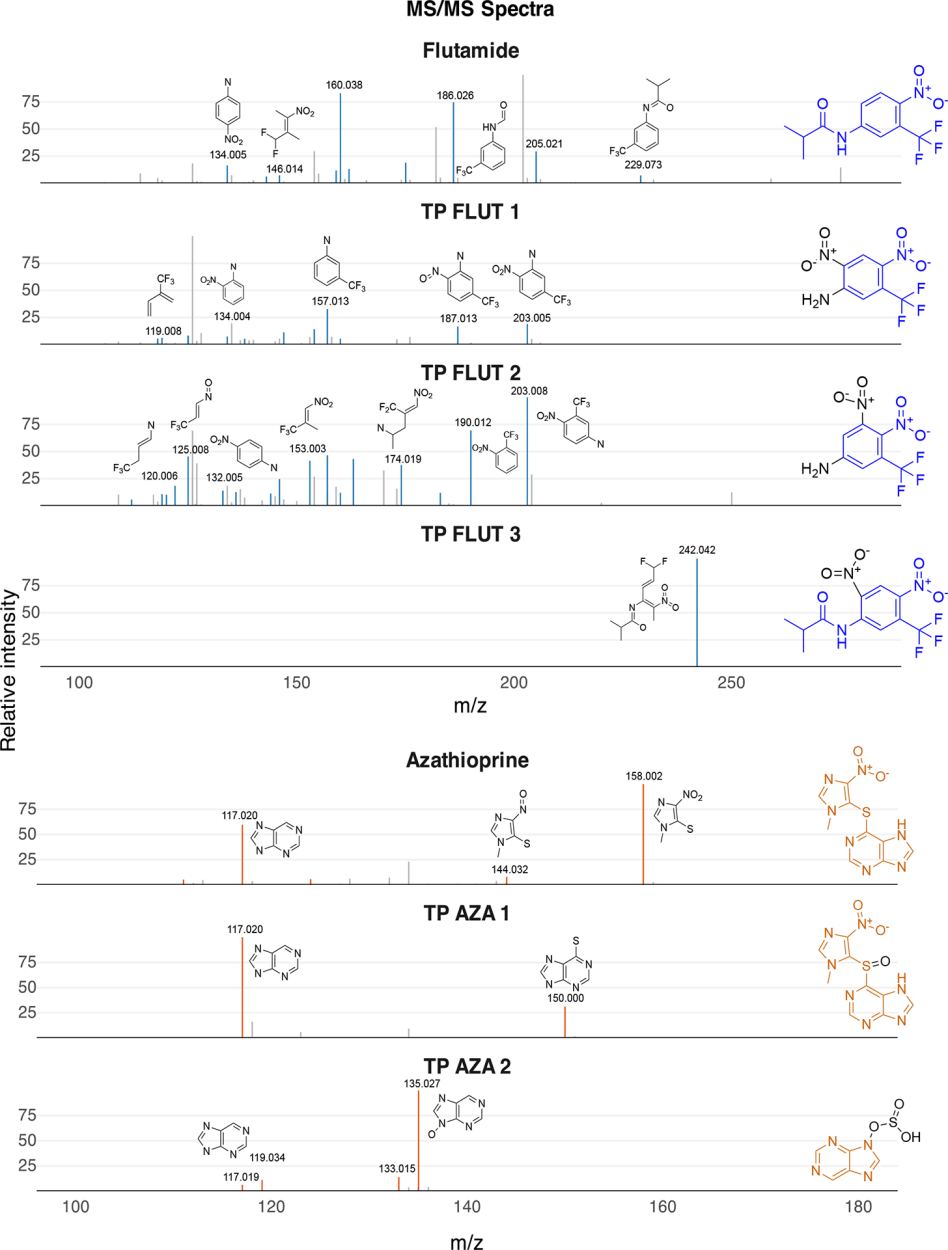
Experimental MS/MS spectra of flutamide (FLUT), azathioprine (AZA), and their transformation products (TP) acquired in negative ESI PASEF mode. The spectra are interpreted by assigning major fragment ions to structurally plausible cleavages and neutral losses, by using MetFrag in silico fragmentation. Colored peaks indicate experimentally observed fragment ions that could be structurally rationalized, while gray peaks represent additional experimental signals for which no structural assignment was proposed. Structures of the parent ions are presented at the right side of the spectra.

TP AZA 2 was not retained on the reversed phase, eluting in the dead volume, but as an exception, it was kept during the data analysis process because its peak area was constantly increasing with ozonation time, providing a clear indication of it being a TP.

When AZA was oxidized, two main TPs were observed. The first, TP AZA 1, formed via oxidation at the sulfur atom, corresponding to mono-oxygenation of AZA. This is consistent with previous studies showing that the sulfur in thioethers is a common site for ozone-elicited oxidation.^35^ At high ozone dosages, a second product, TP AZA 2, appeared. TP AZA 2 exhibited a much lower RT, indicating the formation of a smaller and highly polar sulfur-containing TP formed after cleavage of the nitro-imidazole moiety. Following this cleavage, the sulfur originally present in AZA may be retained on the purine ring and subsequently undergo further oxidation or rearrangement, leading to the formation of AZA TP2, a small polar-sulfonate-type purine TP. A structure where the sulfur is retained was originally considered, although it did not match the adquired MS/MS spectra, which suggested a rearrangement to have occurred. However, the exact nature of the sulfur-containing functionality could not be unambiguously confirmed. Formation of highly oxidized sulfur functionalities, such as sulfonic acids or sulfonates, has been reported during ozonation of sulfur-containing compounds, including biomolecules such as glutathione^36^ and other reduced sulfur species in aqueous systems.^37^ Alternatively, reaction with reactive sulfur-containing species generated in situ, such as hydrogen sulfite (HSO_3_^–^), cannot be excluded.^36^ The MS/MS spectrum of TP AZA 2 was dominated by purine-based fragment ions, and no sulfur-containing fragments were observed, which may reflect preferential loss of the sulfur-containing moiety during fragmentation. Although the imidazole ring could also react with ozone, its reactivity is strongly reduced by the nitro group, which makes the ring less electron-rich.^38^ This could explain why the main reactions occurred at the sulfur instead.

To the best of our knowledge, ozonation of AZA has not yet been reported. However, several studies have investigated its degradation using other AOPs, such as photodegradation.^39^ In that work, the carbon in the purine group was oxidized instead of the sulfur. However, although matching with the observed *m*/*z* value, the MS/MS spectra did not show the characteristic ions from the nitroimidazole group at *m*/*z* 82 (loss of NO_2_) and *m*/*z* 96.^40^ Instead, the predicted MS/MS spectra and CCS values of the feature matched better with TP AZA 1 (Figure 4). Although the proposed structure of AZA TP2 contains sulfur, the observed MS/MS spectrum is dominated by purine-based fragment ions, and no sulfur-containing product ions were detected, suggesting preferential cleavage of the sulfur-containing moiety during fragmentation. Experiments conducted at lower collision energies would be required to provide further confirmation of the structure.

One study has investigated TP formation from FLUT in the solar photo-Fenton process.^24^ The TPs proposed in that study were not detected in any of the samples taken from the ozonation process, indicating that FLUT, similar to AZA, generate different TPs depending on the oxidation treatment. Three TPs were identified, TP FLUT 1 and 2 are rapidly formed and are likely to be structural isomers (similar *m*/*z* and slightly different RT), while TP FLUT 3 appeared later (Figure 3). When analyzed in SIRIUS, both TP FLUT 1 and 2 were classified as dinitroanilines, with the suggested structures matching with the experimental CCS and MS/MS spectra. No realistic structure was found for TP3 in the extensive databases used for annotation. Given that TP1 and TP2 were suggested to be dinitroaniline derivatives and the MS data indicated the addition of one extra nitrogen atom, a target list of potential nitrated FLUT TPs was compiled manually. Integrating this list with predictions from computational tools, FLUT TP3 was identified as the most likely structure among the candidate TPs. However, the signal intensity for TP3 was very low, which may have affected the accuracy of the structural elucidation.

Aromatic nitration has not previously been reported among ozonation-derived transformation products. However, several observations support the likelihood of such TPs. Corona discharge in ambient air is known to generate high levels of NO_*x*_ ^41^ and other reactive nitrogen species.^42^ Even under pure nitrogen, corona discharge can produce aqueous NO_2_ and NO_3_, though the mechanism remains unclear, resulting in aromatic nitrates.^43^ Moreover, ozone-mediated nitration of aromatics via NO_2_ is an established synthetic route, achieving yields of up to 100% for polynitro aromatics.^44^ The nitrogen could also originate from FLUT itself, something that would require isotopically labeled FLUT to prove.

The proposed structure of TP3 also suggest amide hydrolysis, a reaction that cannot be attributed to ozone alone, since molecular ozone does not cleave amide bonds. In contrast, oxygen-based radicals can abstract a hydrogen atom from the α-carbon via a hydrogen atom transfer process, generating an α-carbon radical that reacts with oxygen to form peroxyl or α-hydroxylated intermediates.^45^ This is consistent with previous observations of α-hydroxylation during solar photo-Fenton oxidation of flutamide^24^ and with the main metabolic transformation catalyzed by cytochrome P450.^46^ The resulting α-hydroxyamide is more electrophilic and prone to C–N bond cleavage,^47^ thereby facilitating amide hydrolysis, a reaction also observed in vivo.^46^

### Toxicity Assessment

AZA solution before ozonation showed a nontoxic, even stimulating effect at all concentrations tested. After 120 min of treatment, insignificant inhibition (less than 20%) was recorded at these concentrations. Also, the original FLUT solution and the solution containing TPs after 120 min of ozonation showed insignificant inhibition values (less than 20%) at all tested concentrations. These results indicate that ozonation treatment did not produce any harmful concentrations of TPs.

Since the aim of the study was to experimentally detect possible negative effects of ozonation on the removal of AZA and FLUT using a relatively simple and rapid test on luminescent bacteria, a prediction of the effects of the substances on other important groups of organisms in aquatic ecosystems was performed. Predicted values of toxic effects (LC50/EC50 for the acute toxicity and ChV for the chronic toxicity) for the parent compounds and their TPs, based on the ECOSAR v2.2 predictive model, are shown in Figure 5. Attention should be paid to the fact that predicted values indicate a possible higher toxicity compared to the original substance. For effective protection of aquatic ecosystems, this prediction needs to be verified. However, the predicted higher toxicity of the FLUT TPs aligns with reports that nitroaromatic toxicity often increases with the number of nitro groups.^48^

**Figure 5:**
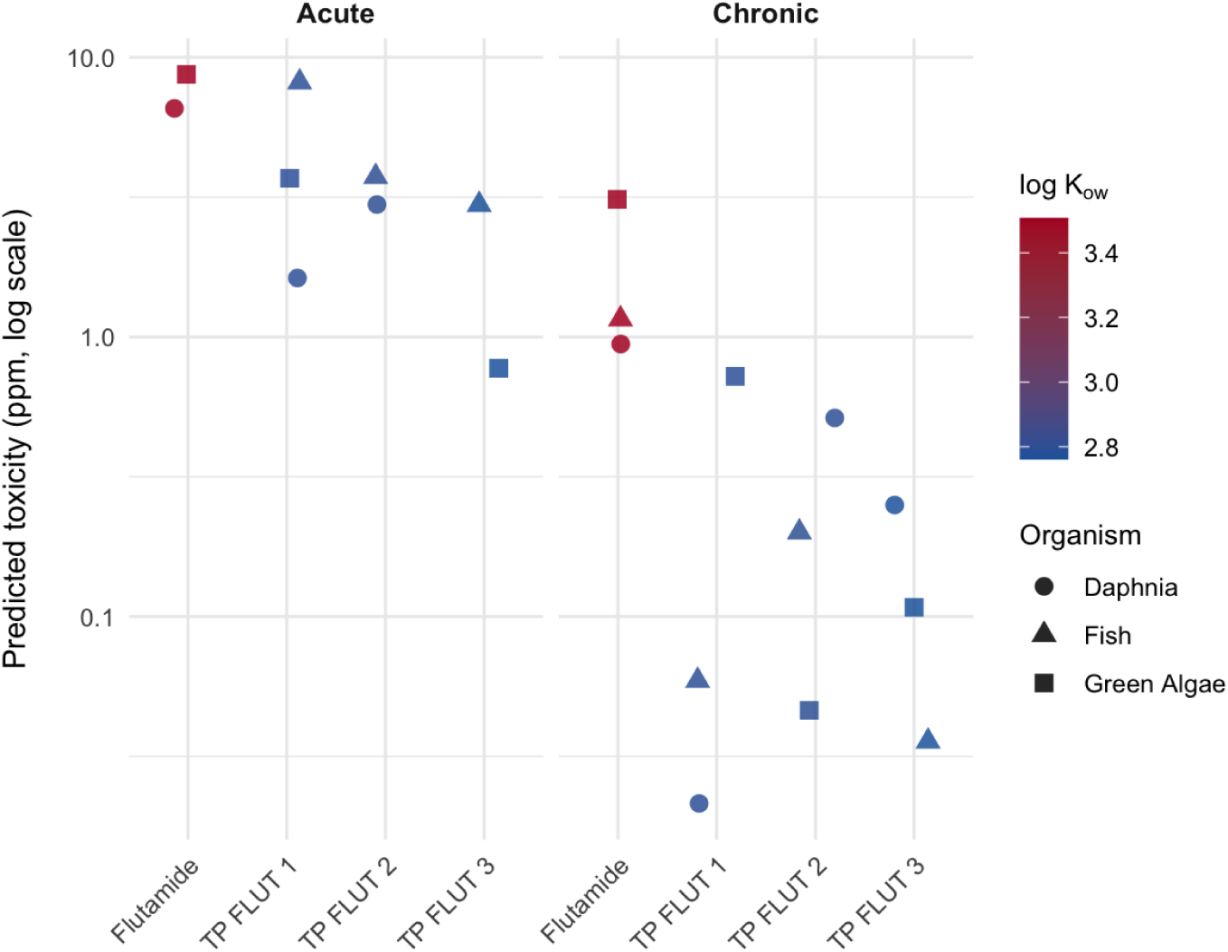
Estimated aquatic toxicity and octanol–water partitioning coefficients (log K_ow_) of flutamide (FLUT) and its tentative transformation products (TPs) using ECOSAR v2.2. Predictions with concentrations exceeding the estimated water solubility are flagged. Predictions above 10^2^ ppm were excluded from the plot. The *y*-axis shows predicted toxicity as LC50/EC50 for acute toxicity and ChV for the chronic toxicity. For azathioprine (AZA) and its TPs values were either above 100 ppm or higher than the predicted solubility.

## Conclusions

For the studied compounds, ozonation transformed both pharmaceuticals, although removal efficiency remained around 50% under the tested conditions. This highlights the need for further development of the process to increase ozone efficiency and/or for its integration with other treatments. A workflow combining suspect screening and de novo prediction with HRMS enabled the detection and structural elucidation of potential TPs for which the toxicological profile could be predicted. Findings of the study underline the need to consider TP’s chronic toxicity in risk assessments of treated waters.

A limitation of the study is the use of reversed-phase chromatography, which restricts the range of detectable compounds, exemplified by TP AZA2 eluting in the void time. Moreover, limitations of current MS/MS libraries reduce annotation confidence, resulting in proposed structures remaining tentative. Consequently, the toxicity predictions from ECOSAR are subject to uncertainty, as they depend on the accuracy of these structural assignments. Experiments were conducted in clean water and not wastewater to focus the investigation on the direct effect of ozone on the selected pharmaceuticals and to facilitate the TPs identification. Whether other TPs would form in real wastewater remains to be investigated. The formation of TPs from reactive sulfur species even under clean conditions highlights the strong influence of the chemical environment on TP formation. Moreover, as ozone from ambient air, which is commonly used in wastewater treatment due to its lower cost, might lead to aromatic nitration, the role of reactive nitrogen species in TP formation must also be considered. Future studies using isotopic labeled flutamide and ozone generated from pure oxygen are needed to verify this reaction. Results show the relevance of further analysis to fill in the existing knowledge gaps on removal efficiency and final toxicity of treated waters.
